# Long‐term memory trajectories in seizure‐free patients following epilepsy surgery for hippocampal sclerosis

**DOI:** 10.1111/epi.18648

**Published:** 2025-10-03

**Authors:** William Alves Martins, Roberta Gomes, Eduardo Leal‐Conceição, Wyllians Vendramini Borelli, Rafael Paglioli, Thomas More Frigeri, Mirna Portuguez, Eliseu Paglioli, Andre Palmini

**Affiliations:** ^1^ Porto Alegre Epilepsy Surgery Program, Neurology and Neurosurgery Services, Hospital São Lucas Pontifical Catholic University of Rio Grande do Sul Porto Alegre Brazil; ^2^ Brain Institute of Rio Grande do Sul Pontifical Catholic University of Rio Grande do Sul Porto Alegre Brazil; ^3^ School of Medicine Pontifical Catholic University of Rio Grande do Sul Porto Alegre Brazil; ^4^ School of Medicine Universidade Luterana do Brasil Canoas Brazil; ^5^ Faculty of Medicine Universidade Federal do Rio Grande do Sul Porto Alegre Brazil

**Keywords:** epilepsy surgery, hippocampal sclerosis, memory, temporal lobe epilepsy

## Abstract

**Objective:**

This study was undertaken to study long‐term memory trajectories over the years in patients with temporal lobe epilepsy and unilateral hippocampal sclerosis (TLE/HS) seizure‐free since surgery.

**Methods:**

This cross‐sectional study included patients with TLE/HS from a single‐center epilepsy surgery program who had been seizure‐free for at least 10 years following anterior temporal lobectomy (ATL) or selective amygdalohippocampectomy (SAH). Memory performance was evaluated preoperatively (T1), 1–4 years postoperatively (T2), and 10–15 years after surgery (T3). Logistic regression evaluated variables correlated with memory function at each point in time. A reliable change index was performed to identify changes in individual measures.

**Results:**

A total of 54 patients were included, of whom 36 (66%) were male and 52 (96%) right‐handed. Patients with left HS and normal preoperative Rey Auditory Verbal Learning Test or Wechsler Memory Scale–Revised (WMS‐R) logical memory showed worsening at T2 (13% × 52%, *p* = .029; 0 × 31%, *p* < .015, respectively) and T3 (27% × 63%, *p* = .045; 22% × 81%, *p* < .001, respectively). Visual reproduction (WMS‐R) following nondominant surgery also deteriorated at T3 for patients who improved or sustained normal performance between T1 and T2 (33% × 50%, *p* = .64). The predictive factors for memory decline included normal preoperative memory function (odds ratio [OR] = 15, 95% confidence interval [CI] = 4.03–55.9, *p* < .001 for logical memory; OR = 1.5, 95% CI = 1.12–2.01, *p* = .007 for visual reproduction), younger age (OR = 1.2, 95% CI = 1.12–1.28, *p* < .001), dominant‐side surgery (OR = 3.66, 95% CI = 1.49–8.95, *p* < .01), and lower education level (OR = 8.74, 95% CI 1.77–43.2, *p* = .008). The SAH technique was associated with better long‐term verbal learning outcomes compared to ATL (OR = 3.02, 95% CI = 1.17–7.81, *p* = .02).

**Significance:**

Memory preservation or improvement in the first few postoperative years is usually not sustained in the long term, suggesting that disease progression surpasses plasticity over the years.

**Key points**

- Long‐term memory trajectories after epilepsy surgery for TLE/HS show a tendency toward delayed decline, even in seizure‐free patients.
- Normal preoperative memory scores, surgery on the dominant (usually left) hemisphere, younger age, and lower education were associated with higher risk of long‐term verbal memory worsening.
- SAH was associated with better long‐term verbal learning outcomes than ATL.
- RCI analyses confirmed that most patients did not experience significant memory changes over time, but decline was more common than improvement.
- Initial postoperative memory preservation or improvement often did not persist at long‐term follow‐up (≥10 years), highlighting the need for lifelong cognitive monitoring.

## 1 INTRODUCTION

The possibility of functional deficits following epilepsy surgery significantly influences surgical candidacy and the extent of resection.^1^, ^2^ Anteromesial temporal lobe epilepsy (TLE) surgery is typically safe, but the potential for significant memory decline must be considered. Several predictors, particularly the preresection structural and functional status of these areas as assessed by magnetic resonance imaging (MRI) and neuropsychological evaluations, have been identified.^3^ Generally, better preserved structure and function are associated with higher risks of postoperative memory deficits.^1^, ^2^, ^4^

For patients with TLE and hippocampal sclerosis (TLE/HS) who already exhibit preoperative memory issues, the risk of additional memory decline is considered small.^4^ However, most postoperative memory assessments are conducted within the first few years, whereas long‐term function is seldom addressed. Furthermore, grouping patients with different TLE pathologies can confound outcome assessments.^5^, ^6^ A test–retest setting can be influenced by practice effects, potentially inflating scores.^7^ To mitigate this, a method using reliable change values was developed to distinguish true alterations from repetition bias.^8^

This study examines individual performance on standardized memory tests before surgery and at two postoperative intervals (1–4 years and 10–15 years) in patients with confirmed HS who have been seizure‐free for >1 decade. Postoperative memory performance varies according to preoperative function in the early years and stabilizes in the long term.^1^, ^5^, ^9^ We also explore the idea that mechanisms of functional reorganization may be activated during epilepsy, potentially stabilizing or improving memory performance postoperatively. Nevertheless, if TLE/HS is associated with broader pathological progression affecting large cortical and subcortical networks, long‐term memory function could worsen despite seizure control.^10^ In this study, early and long‐term neuropsychological evaluations were performed on seizure‐free subjects who underwent surgery for TLE/HS, using an individual reliable change index (RCI) to assess changes accurately.

## 2 MATERIALS AND METHODS

In this cross‐sectional single‐center study, 108 patients who underwent surgery for TLE/HS in the Porto Alegre Epilepsy Surgery Program were screened for participation. Twenty‐three patients were excluded due to seizure recurrence; one patient had died and another 26 were lost to follow‐up. Four patients did not consent. Fifty‐four patients were included in the final analysis. All had been seizure‐free for at least 10 years since either anterior temporal lobectomy (ATL) or selective amygdalohippocampectomy (SAH)^11^, ^12^ and had a mean follow‐up of 15.5 years (SD = 3.4).^13^

Interictal spikes were classified as having >90% or <90% unilateral predominance. Thirty‐four patients (63%) were operated on the left side, and 29 (54%) underwent SAH. Electroencephalography (EEG), MRI, pathology, and surgical techniques have been detailed elsewhere.^12^ Outcomes were assessed independently by the neurological and neurosurgical teams, and all have been Engel class IA since surgery.^14^ Attempts were made to maintain therapeutic dosages of a first‐line antiseizure drug (ASD) for at least 2 years after surgery. Detailed demographic data are presented in Table 1.

**TABLE 1:** Demographic and clinical data (total sample, *N* = 54).

Variable	*n*	%
Sex		
Female	18	33.3
Male	36	66.7
Age, years		
Mean ± SD (range)	49.8 ± 8.4	(28.0–64.0)
Median (1st–3rd quartile)	51.0	(45.7–56.0)
Schooling		
Elementary school	28	51.9
High school	14	25.9
College	12	22.2
Marital status		
Single	21	38.9
Married	29	53.7
Separated/divorced	3	5.6
Widowed	1	1.9
Handedness		
Right	52	96.3
Left	2	3.7
Age at onset of epilepsy, years		
Mean ± SD (range)	8.2 ± 8.7	(.2–35.0)
Median (1st–3rd quartile)	4.0	(1.0–15.3)
Presurgical EEG		
Unitemporal	41	76
Bitemporal	13	24
Duration of epilepsy, years [MD = 1]		
Mean ± SD (range)	26.2 ± 10.5	(5–46)
Median (1st–3rd quartile)	25.0	(18.0–34.3)
Operated hemisphere		
Left	34	63.9
Right	20	37.1
Type of surgery		
ATL	25	46.3
SAH	29	53.7
Age at surgery, years		
Mean ± SD (range)	34.3 ± 8.4	(16–47)
Median (1st–3rd quartile)	35.0	(29.7–39.0)
Retest interval T1/T2, years		
1	5	9.3
2	15	27.8
3	16	29.6
4	18	33.3
Age at T2, years		
Mean ± SD (range)	37.2 ± 8.4	(18–50)
Median (1st–3rd quartile)	38.0	(32.7–42.3)
Antiseizure drugs		
No	30	55.6
Yes	24	44.4
Time to last evaluation (T3)		
Mean ± SD (range)	15.5 ± 3.4	(10.0–22.0)
Median (1st–3rd quartile)	14.0	(13.0–19.0)
Paid occupation		
Yes	33	61.1
No	21	38.9

Abbreviations: ATL, anterior temporal lobectomy; EEG, electroencephalography; MD, missing data; SAH, selective amygdalohippocampectomy; T1, presurgical period; T2, postsurgical period: 1–4 years; T3, postsurgical period: ≥10 years.

### 2.1 Neuropsychological evaluation

Verbal memory was assessed with the Wechsler Memory Scale–Revised (WMS‐R) logical memory–delayed recall subtest, and visual memory was assessed with the WMS‐R visual reproduction–delayed recall subtest.^15^ Verbal learning was assessed with the Rey Auditory Verbal Learning Test (RAVLT) total learning (sum of trials 1–5),^16^ and scored from the sum of the number of words remembered immediately following each of five oral presentations of a 15–word list.^17^ All neuropsychological scores (WMS‐R) were analyzed using age‐corrected normative scores. For score interpretation, we used the Brazilian adaptation of the WMS‐R and Brazilian normative data for the RAVTL.^15^, ^16^

Patients were evaluated at three timepoints: preoperatively (T1), 1–4 years after surgery (T2), and ≥10 years after surgery (T3). All evaluations used the same tools in the three timeframes (WMS‐R and RAVTL). Range, mean (SD), and median (interquartile range [IR]) ages at T1, T2, and T3, as well as time elapsed between each two evaluations, are shown in Table 2. According to the standardization proposed by Wechsler, we considered significantly abnormal in each test those scores inferior to the threshold of 1 SD below the mean score (i.e., < −1 SD).

**TABLE 2:** Age at each evaluation and time elapsed between evaluations (total sample, *N* = 54).

Variable	Value, years
Age at T1	Mean ± SD: 34.3 ± 8.4 Median: 35.0 (29.7–39.0)
Age at T2	Mean ± SD: 37.2 ± 8.4 Median: 38.0 (32.7–42.3)
Age at T3	Mean ± SD: 49.8 ± 8.4 Median: 51.0 (45.7–56.0)
Time between T1 and T2	Mean ± SD: 2.9 ± .9 Median: 3.0 (2.0–4.0)
Time between T2 and T3	Mean ± SD: 12.7 ± 3.1 Median: 11.5 (10.0–15.0)
Time between T1 and T3	Mean ± SD: 15.5 ± 3.4 Median: 14.0 (13.0–19.0)

*Note*: Median values are followed by interquartile range (25%–75%).

Abbreviations: T1, presurgical period; T2, postsurgical period: 1–4 years; T3, postsurgical period: ≥10 years.

In addition, we assessed absolute changes in scores of >1 SD between two evaluations, analyzing postoperative memory changes in two ways. First, we considered crossing the −1 SD threshold (criterion 1) with three possibilities: (1) improvement: scores below −1 SD at T1 but higher at T2 or T3; (2) worsening: scores within normal limits at T1 but below −1 SD at T2 or T3; and (3) no significant variation: scores remaining on the same side of the threshold across evaluations. Second, we analyzed memory variation according to absolute changes in scores by >1 SD (criterion 2) with three possibilities: (1) improved: scores increased by >1 SD; (2) worsened: scores decreased by >1 SD; and (3) no significant change: scores varied by <1 SD. Performance at T1 was evaluated only by criterion 1. Improvement and no variation were grouped as no worsening, that is, favorable memory outcome. We defined scores < −1 SD of normative means as “abnormal” to increase sensitivity to subtle memory changes.

At T2, patients were either at the same dosage or beginning reduction of ASDs. Before surgery, 14 patients (26%) used one, 31 (57%) used two, and nine (17%) used three or more ASDs. At T3, 30 patients (56%) had discontinued medication (*p* < .001). Beck inventories were used to probe depressive (Beck Depression Inventory) and anxiety (Beck Anxiety Inventory) symptoms, and quality of life was evaluated through the Quality of Life ‐ 65 (QoL 65).^18^

### 2.2 Reliable change index

The RCI was used to minimize biases and measure reliable change in scores at an individual level. The formula used was:

$$\begin{array}{c} \mathrm{RCI}=\left(\left(T2-T1\right)-\left(M2-M1\right)\right)/\mathrm{SED}\\ \mathrm{RCI}=\left(\left(T3-T2\right)-\left(M3-M2\right)\right)/\mathrm{SED}\\ \mathrm{RCI}=\left(\left(T3-T1\right)-\left(M3-M1\right)\right)/\mathrm{SED} \end{array}$$

where T1 is the preoperative memory score, T2 is the early postoperative score (1–4 years), and T3 is the late postoperative score (≥10 years); M1 is the group mean preoperative score (T1); M2 is the group mean early postoperative score (T2); M3 is the group mean late postoperative score (T3); and SED is the standard error of a difference. The difference between time periods (T1, T2, T3) measures the individual change, whereas the difference of changes (M1, M2, M3) measures the mean of the group in the memory tests of the WMS‐R and RAVTL. We used normative samples of the group divided by age.

An average improvement in the test performance in the normative sample (M2 − M1 > 0, M3 − M2 > 0, and M3 − M1 > 0) is interpreted as a practice effect on a group level; hence, the individual change is corrected for the average practice effect with (T_x_ − T_x−1_) − (M_x_ − M_x−1_). The SED details the dispersion of change scores attributable to the instability of measures, defined as the SD of the mean observed difference score using the formula SED = (2 × SEM^2^)^1/2^ where SEM = SD ([1 − r]^1/2^), SD refers to preoperatory scores (T1), and r is the reliability coefficient. RCI cutoff scores for these measures were calculated as ±1.645 (*p* < .1, two‐tailed). We therefore entered the RCI as an independent criterion. We selected a 90% confidence interval (CI; ±1.645) for RCI cutoff values to adopt a more conservative criterion, reducing the risk of false positives (type I errors) in the context of multiple repeated assessments. Although an 80% CI (±1.28) is more sensitive and commonly applied in neuropsychological research, we considered that our relatively small sample size and long‐term follow‐up could increase the probability of spurious findings.

The study was approved by our institutional review board and adhered to national standards for research involving human subjects. All patients provided written informed consent prior to their inclusion in the study.

### 2.3 Statistical analysis

Continuous variables were described using mean, median, SD, and IR. Categorical variables were presented as absolute and relative frequencies. Continuous variables were compared using Student *t*‐test or Mann–Whitney test as appropriate. Linearity was evaluated using Spearman correlation coefficient. For comparisons among three or more independent groups, one‐way analysis of variance was performed with post hoc comparisons using Scheffe test. Fisher exact test was used to evaluate categorical variables describing the relationship between baseline memory function and postoperative performance. Preoperative and postoperative memory test scores (WMS‐R and RAVTL) and the RCI were analyzed using RStudio (v1.0.136). Predictive factors for postoperative memory performance evolution were identified using a univariate Poisson regression model. Variables with *p* < .10 in the univariate analysis were included in the multivariate analysis, and the odds ratio (OR) with a 95% CI was calculated. Significance was set at *p* < .05. Statistical Package for the Social Sciences (SPSS Inc., 2011) version 20.0 was also used for statistical analysis.

## 3 RESULTS

A total of 54 individuals were included, of whom 36 were male and 52 (96%) right‐handed. The median age at seizure onset was 4 years (IR = 1–15.3), and the median duration of epilepsy was 25 years (IR = 18–34.3). Only seven individuals had a history of recurrent tonic–clonic generalized seizures. The age at surgery ranged from 16 to 47 years (mean = 34.3, SD = 8.4), and the age at the last follow‐up ranged from 28 to 64 years (mean = 49.3, SD = 8.4). Roughly half (52%) had only primary education, and 33 patients (61%) were currently working.

**FIGURE 1:**
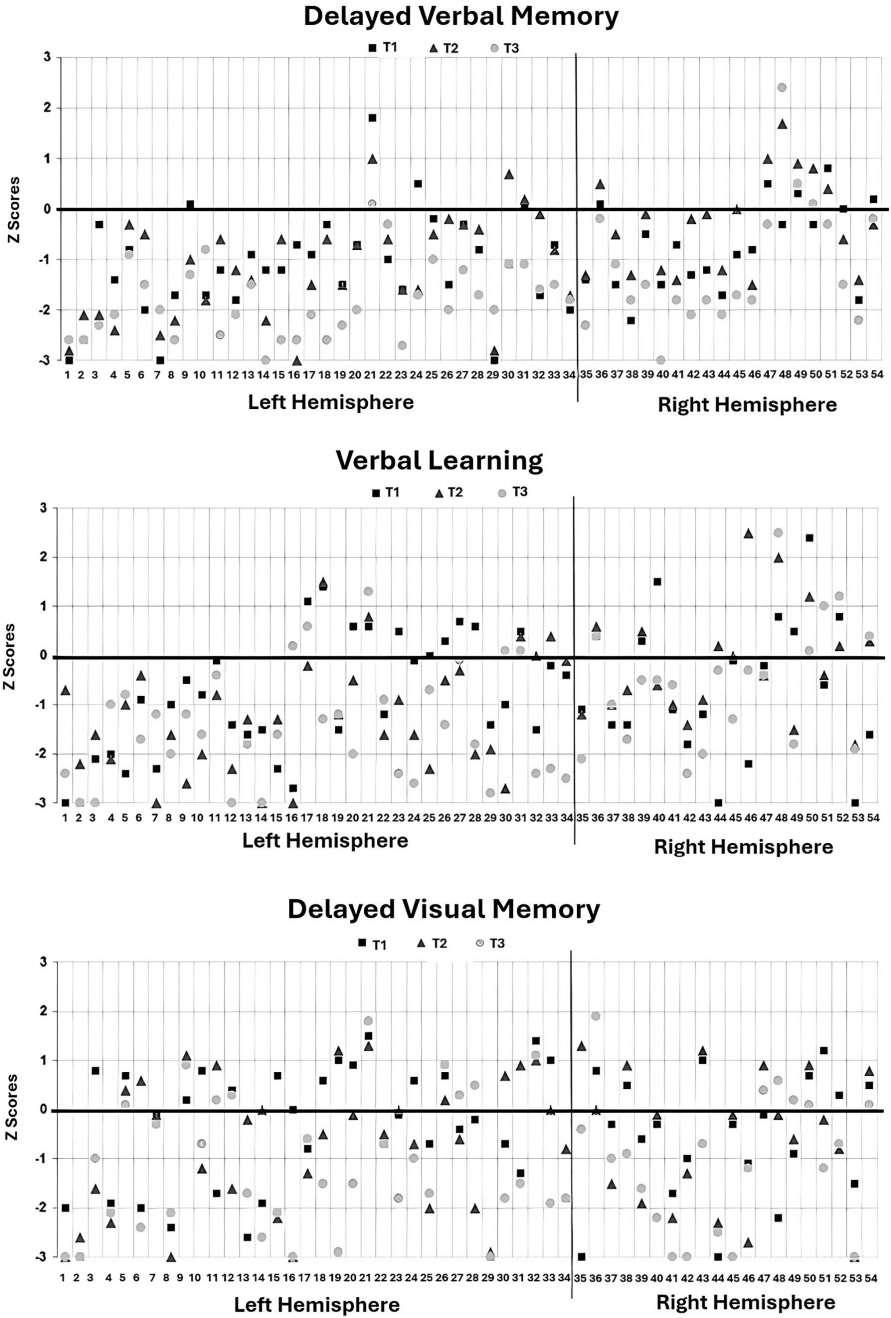
Memory trajectory of each patient according to *Z*‐score at each timepoint.

Nondominant hemisphere patients fared significantly better than those operated on the left in logical memory (WMS‐R), at both T2 (−.29 ± .95 vs. −1.18 ± 1.03, *p* = .002) and T3 (−1.14 ± 1.26 vs. −1.81 ± .73, *p* = .013), and also in RAVLT (−.17 ± 1.14 vs. −1.34 ± 1.40, p = .002 at T2; −.56 ± 1.26 vs. −1.52 ± 1.22, *p* = .008 at T3). Memory scores at each timepoint according to the side of resection are detailed in Table 3 and illustrated in Figure 1. Effect sizes were consistently moderate to large. For example, in the comparison of side of resection, patients with right HS showed better outcomes in WMS‐R logical memory delayed recall (T2: d = .89, T3: d = .70) and RAVTL total learning (T2: d = .89, T3: d = .78). Risk ratios (RRs) also demonstrated increased vulnerability among patients with normal preoperative scores, particularly for left HS cases in logical memory (T3 worsening: RR = 3.66, 95% CI = 1.49–8.95) and RAVTL (T2 worsening: RR = 3.95, 95% CI = 1.01–15.36).

**TABLE 3:** Demographic, clinical, and neuropsychological data by side of hippocampal sclerosis.

Category	Left [*n* = 34], mean (SD)	Right [*n* = 20], mean (SD)	*p*
Age at surgery, years	33.1 (9.6)	36.5 (5.4)	NS
Age of onset, years	6.7 (7.7) [MD = 3]	10.3 (10.1) [MD = 8.5]	NS
Interval T1–T3, years	15.1 (3.4)	16.1 (3.4)	NS
WMS‐R logical memory–delayed recall [T1]	−1.13 (1.02)	−.71 (.85)	.128
RAVTL total learning [sum of trials 1–5; T1]	−.89 (1.32)	−.75 (1.75)	.798
WMS‐R visual reproduction–delayed recall [T1]	−.55 (1.51)	−.57 (1.28)	.944
WMS‐R logical memory–delayed recall [T2]	−1.18 (1.03)	−.29 (.95)	.002
RAVTL total learning [sum of trials 1–5; T2]	−1.34 (1.40)	−.17 (1.14)	.002
WMS‐R visual reproduction—delayed recall [T2]	−.79 (1.53)	−.59 (1.47)	.637
WMS‐R logical memory—delayed recall [T3]	−1.81 (.73)	−1.14 (1.26)	.013
RAVTL total learning [sum of trials 1–5; T3]	−1.52 (1.22)	−.56 (1.26)	.008
WMS‐R visual reproduction–delayed recall [T3]	−1.25 (1.50)	−1.16 (1.46)	.872

Abbreviations: MD, median; RAVTL, Rey Auditory Verbal Learning Test; T1, presurgical period; T2, postsurgical period: 1–4 years; T3, postsurgical period: ≥10 years; WMS‐R, Wechsler Memory Scale–Revised.

The following section analyzes results according to the side of the mesial temporal sclerosis (MTS).

### 3.1 Patients with left‐sided MTS

#### 3.1.1 WMS‐R logical memory–delayed recall

Eighteen patients (52%) had abnormal WMS‐R logical memory–delayed recall scores before surgery. Six of these patients (33%) showed significant improvement in the short term (T2), but all returned to baseline (T1) levels by T3. None worsened in the first year (T2), and only four of the 18 worsened by >1 SD at T3. Among the 16 patients who had normal logical memory (WMS‐R) scores at T1, five (31%) significantly worsened at T2 (*p* < .015). The remaining 11 (69%) maintained normal performance at T2, but only three had normal scores at T3. When comparing preoperative scores to the last evaluation (T3 vs. T1), only 22% of those with abnormal logical memory (WMS‐R) scores worsened at T3, compared to 81% of those with normal scores (*p* = .001). Only one patient (3%) significantly improved logical memory (WMS‐R) scores between T2 and T3.

Overall, only three of the 16 patients (19%) with normal preoperative logical memory (WMS‐R) did not worsen in the long term. Additionally, among the 17 patients with significant dysfunction at T2, only one worsened further at T3, in contrast to 76% of those with normal scores at T2 (*p* = .0001).

#### 3.1.2 RAVLT total learning (sum of trials 1–5)

Fifteen patients (44%) had abnormal RAVLT scores before surgery. Only two of these (13%) worsened in the first year, compared to 10 of 19 patients (52%) with normal scores at T1 (*p* = .029). The remaining nine of these 19 patients (48%) maintained normal performance at T2, although only four sustained normal performance at T3. Four of the 15 patients (27%) with abnormal scores at T1 significantly improved at T2, but two of them deteriorated by T3. Additionally, of the 15 patients with dysfunctional scores at T1, only four (27%) worsened further at T3, compared to 12 of 19 (63%) who had normal function before surgery (*p* = .045). Overall, only seven of 19 patients (37%) with normal preoperative RAVLT function did not worsen in the long term. Five patients (15%) significantly improved their RAVLT scores between T2 and T3.

#### 3.1.3 Visual reproduction (WMS‐R) delayed recall

Eleven patients (32%) had abnormal scores on visual reproduction before surgery. One (9%) worsened in both the short and long term, compared to nine of 23 (39%) who had normal scores at T1 (*p* = .11). Of the 23 patients with normal scores at T1, 14 (61%) maintained normal performance at T2, although six worsened by T3. Eight of the 11 patients (73%) with abnormal scores significantly improved at T2, but five of these worsened again by T3. None of the 13 patients with significant dysfunction at T2 worsened further by T3, compared to 11 of the 21 (52%) who had normal scores at T2 (*p* = .001). Five patients (15%) significantly improved visual reproduction (WMS‐R) scores between T2 and T3.

The RCI for logical memory (WMS‐R), RAVLT, and visual reproduction scores showed mixed findings based on the timepoint and type of surgery performed on patients with left HS. A total of 126 RCIs were calculated for ATL patients and 177 RCIs for SAH patients. Among these, 116 (92%) ATL patients and 156 (88.1%) SAH patients showed no change between T1–T2, T2–T3, and T1–T3. For ATL patients, one showed a decreased RCI in T1–T2, one showed improvement in T2–T3 and T1–T3, and 13 remained unchanged across all timepoints. Among the 20 SAH patients, two showed improved RCI, two showed decreased RCI, and 16 remained unchanged between T1–T2 and T2–T3. In T1–T3, one patient showed improvement, two decreased, and 17 remained unchanged.

For RAVLT scores, among the 14 ATL patients, only one improved, one decreased, and 12 remained unchanged between T1 and T2. There were no reliable changes in RAVLT scores between T2 and T3. Between T1 and T3, one decreased and 13 remained unchanged. Among the 20 SAH patients, the RCI for RAVLT decreased in three subjects and remained unchanged in 17 between T1 and T2. Between T2 and T3, two subjects showed improvement, one decreased, and 17 remained unchanged. From T1 to T3, one improved and 19 remained unchanged.

For WMS‐R visual reproduction–delayed recall scores in ATL patients, one decreased and 13 remained unchanged between T1 and T2. Between T2 and T3, one improved, one decreased, and 12 remained unchanged. From T1 to T3, one decreased and 13 remained unchanged. Among SAH patients, two improved and 18 remained unchanged between T1 and T2. Between T2 and T3, one improved, one decreased, and 18 remained unchanged. From T1 to T3, two decreased and 18 remained unchanged.

### 3.2 Patients with right‐side MTS

#### 3.2.1 WMS‐R logical memory–delayed recall

Eight patients (40%) had abnormal scores on logical memory (WMS‐R) before surgery. Three of these (37.5%) significantly improved performance in the short term (T2), but all deteriorated to baseline at T3. None worsened in the short term, in comparison to two of the 12 (17%) who presented normal scores at T1 (*p* = .49). Furthermore, of the eight patients who had dysfunctional scores at T1, only one (12.5%) additionally worsened at T3, in contrast to six of 12 (50%) who had normal scores before operation (*p* = .16). Thus, only half the patients with normal logical memory (WMS‐R) did not worsen in the long term following a nondominant temporal lobe resection. No patient significantly improved logical memory (WMS‐R) scores between T2 and T3.

#### 3.2.2 RAVLT total learning (sum of trials 1–5)

Half of the 20 patients had abnormal scores on RAVLT before surgery. Eight of these (80%) significantly improved performance in the first years (T2), and five sustained such improvement at T3. Only three patients (30%) with normal performance at T1 worsened in the short term (*p* < .21), and four (40%) showed abnormal results at T3 (*p* = .08). No patient significantly improved RAVLT scores between T2 and T3.

#### 3.2.3 Visual reproduction (WMS‐R) delayed recall

Six patients (30%) had abnormal scores on visual reproduction (WMS‐R) before surgery. Two (10%) worsened early on, compared to five (36%) who had normal scores at T1 (*p* > .99). Furthermore, of the six patients who had dysfunctional scores at T1, two (33%) worsened at T3, compared to seven (50%) who had normal performance before surgery (*p* = .64). Eight patients worsened from T2 to T3 (*p* = .64). Only one patient significantly improved visual reproduction (WMS‐R) scores between T2 and T3. Of note, two patients (33%) with dysfunctional scores at T1 significantly improved performance in the short term (T2) and sustained such improvement at T3.

#### 3.2.4 RCI in relation to surgery in right HS

Overall, RCI scores of individuals with right HS remained largely similar between timepoints and types of surgery. A total of 99 RCIs were assessed for ATL patients, and 81 RCIs were calculated for SAH. Only four showed reliable improvement in at least one memory test (two in RAVLT and two in visual reproduction), whereas eight (9.8%) subjects in the SAH group showed increased memory scores (three in logical memory, three in RAVLT, and two in visual reproduction).

Several variables were associated significantly with memory trajectories (Table 4). Risk of worsening of logical memory (WMS‐R) or RAVLT between preoperative evaluation (T1) and the first postoperative years (T2) was significantly increased by normal preoperative function (OR = 15, 95% CI = 4.03–55.9, *p* < .001 for logical memory [WMS‐R]; OR = 1.5, 95% CI = 1.12–2.01, *p* = .007 for visual reproduction), young age (OR = 1.2, 95% CI = 1.12–1.28, *p* < .001), surgery on the dominant side (OR = 4.97, 95% CI = 1.89–13.1, *p* < .001), and low schooling (OR = 8.74, 95% CI = 1.77–43.2, *p* = .008). Furthermore, a < 90% unilateral predominance of interictal spikes showed a trend for worse outcomes (OR = 3.86, 95% CI = .94–15.8, *p* = .06). Functional worsening between the first postoperative years (T2) and the long term (T3) in all memory domains was significantly associated with normal preoperative scores (OR = 2.42, 95% CI = 1.38–4.26, *p* = .002), low schooling levels (OR = 2.65, 95% CI = 1.04–6.71, *p* = .041 for RAVLT; OR = 6.07, 95% CI = 1.87–19.7, *p* = .003 for visual reproduction), surgery in the dominant side (OR = 2.78, 95% CI = 1.09–7.07, *p* = .032), and nonselective surgical technique (OR = 3.02, 95% CI = 1.17–7.81, *p* = .02). Tables 4 and 5 show all risk factors for memory worsening from T1 to T2 and T2 to T3, respectively.

**TABLE 4:** Variables associated with worsening of memory function between T1 and T2.

Memory domain	Regression coefficients T1–T2		
	OR	95% CI	*p*
WMS‐R logical memory–delayed recall			
EEG (bitemporal)	3.86	.94–15.8	.060
Age at operation	.83	.78–.89	<.001
Schooling (elementary school)	8.74	1.77–43.2	.008
Basal T1	15.0	4.03–55.9	<.001
RAVTL–total learning (sum of trials 1–5)			
Operated hemisphere (left)	4.97	1.89–13.1	.001
Basal T1	2.29	1.44–3.64	<.001
WMS‐R visual reproduction–delayed recall			
Basal T1	1.50	1.12–2.01	.007

Abbreviations: CI, confidence interval; EEG, electroencephalography; OR, odds ratio; RAVTL, Rey Auditory Verbal Learning Test; T1, presurgical period; T2, postsurgical period: 1–4 years; WMS‐R, Wechsler Memory Scale–Revised.

**TABLE 5:** Variables associated with worsening of memory function between T2 and T3.

Memory function	Regression coefficient T2–T3		
	OR	95% CI	*p*
WMS‐R logical memory–delayed recall			
Operated hemisphere (left)	2.78	1.09–7.07	.032
Basal T2	2.42	1.38–4.26	.002
RAVTL total learning (sum of trials 1–5)			
Type of surgery (ATL)	3.02	1.17–7.81	.022
Schooling (elementary school)	2.65	1.04–6.71	.041
Basal T2	3.54	1.94–6.44	<.001
WMS‐R visual reproduction–delayed recall			
Schooling (elementary school)	6.07	1.87–19.7	.003
Basal T2	2.17	1.39–3.40	.001

Abbreviations: ATL, anterior temporal lobectomy; CI, confidence interval; OR = odds ratio; RAVTL, Rey Auditory Verbal Learning Test; T2, postsurgical period: 1–4 years; T3, postsurgical period: ≥10 years; WMS‐R, Wechsler Memory Scale–Revised.

### 3.3 Contralateral functional reserve in patients receiving surgery on the left side

Among the 17 patients who received surgery on the left side who experienced long‐term (T3) verbal memory decline, four (23%) had abnormally low scores in visual reproduction at first evaluation, compared to seven of the other 17 patients (41%) who did not worsen at T3 (*p* = .46). Likewise, six (37%) patients who had surgery on the left and worsened on RAVLT at T3 also presented low scores in visual reproduction compared to five of the other 18 (28%) who did not worsen at T3 (*p* = .71).

## 4 DISCUSSION

This long‐term prospective study demonstrates that memory trajectories follow a nonuniform trajectory, with a tendency to worsen over time, irrespective of seizure freedom after temporal lobe resection. Memory outcome remains an upmost concern prior to surgical decision in TLE/HS. Although the procedure is highly effective for seizure control,^11^, ^13^ there is a risk of inducing significant memory abnormalities, particularly early on. Therefore, anticipating memory performance after unilateral resection of temporal lobe structures is paramount to presurgical counseling. Although substantial research exists on short‐term memory outcomes, there is a scarcity of data addressing long‐term memory performance, particularly ≥10 years after temporal lobe surgery.^1^, ^6^, ^19^, ^20^ Most studies have shown either performance plateauing a few years after surgery or some the long‐term decline.^6^ Most only compare pre‐ and postoperative performances using absolute scores without determining a threshold for normal versus abnormal function.^4^, ^5^, ^21^, ^22^ Furthermore, most studies include patients with variable seizure outcomes and different etiologies of TLE, complicating the analysis of long‐term memory outcomes in seizure‐free patients with TLE/HS.^2^, ^3^, ^4^, ^23^

In our study, dominant HS (left) patients with normal preoperative scores in verbal learning and memory maintained these scores in 69% of cases during the first year postsurgery, whereas only 18% did so in the long term. Approximately 30% of those with abnormal scores at T1 significantly improved at T2, but these improvements were transitory. These findings suggest two mechanisms of functional reorganization: one sustaining or recovering function shortly after surgery and another likely related to disease progression or network instability affecting long‐term function.^10^, ^24^ The small number of patients showing improvement between T2 and T3 suggests the relevance of disease progression for functional outcomes, contrasting with reports of memory function stabilization a few years postsurgery and underscoring the importance of long‐term follow‐up.^1^, ^19^ Of course, these results must be interpreted in the context of the natural history of cognitive changes in temporal lobe epilepsy. Specifically, it is well known that memory functions do worsen over time even in patients who do not have surgery.^6^, ^25^, ^26^ herefore, the stability of memory performance in the first years following successful operation is a significant gain for these people, even if memory circuits cannot sustain such performance in the long term ‐ an inevitable consequence of TLE. Because all patients are seizure‐free, this delayed worsening is unlikely to be due to recurrent seizures.

Nondominant right‐side HS patients displayed abnormal preoperative logical memory (WMS‐R) scores in 40% of cases. Most patients with normal preoperative scores did not worsen in the first postoperative years, and fewer experienced long‐term decline, suggesting a lesser issue with verbal memory or learning decline in these patients.^12^, ^23^ Despite known material‐specific memory patterns,^27^, ^28^ only 30% of patients with right (nondominant) HS had abnormal visual reproduction scores preoperatively, with most maintaining normal function within the first years. Long‐term results showed that 40% did not maintain function, even those who had initially improved in logical memory (WMS‐R).

The evolution of verbal learning and memory was influenced by preoperative performance, age, education level, surgical technique, and distribution of EEG spikes. Preoperative performance strongly predicted postoperative evolution, both in the first few years and in the long term.^22^, ^29^, ^30^ Lower education levels likely reflect reduced plasticity ability, related to impaired cognitive reserve.^6^ Bitemporal preoperative EEG spikes displayed a slightly higher risk of functional decline. Such discharges have been shown to lower chances of Engel class IA outcomes significantly, without not affecting class I outcome overall.^12^, ^13^ Whether these discharges interfere with pre‐ and postoperative reorganization of memory circuits should be further explored. Contrary to expectation, older age was associated with less functional decline, indicating that reorganization mechanisms are likely more complex.^2^, ^4^, ^17^, ^19^, ^31^ Finally, a selective surgical technique (SAH) was associated with a significantly lower risk of decline in RAVLT, particularly in patients who either improved or sustained a good performance in the first years after operation. This finding suggests that preserving neocortical structures around a sclerotic hippocampus contributes to long‐term cognitive outcomes,^12^ although it may be associated with a lower chance of seizure freedom.^32^ The balance between memory preservation and seizure freedom still dominates the debate, as less invasive strategies are employed despite being less effective.^33^

Our study provides valuable insights into the long‐term memory outcomes of patients undergoing TLE surgery but has limitations. One significant limitation is the relatively small sample size of 54 patients, which may impact generalizability. The observational design and lack of a control group limit our ability to attribute memory changes solely to the surgery, as other factors such as ongoing medical management and disease progression may also play a role. Cognitive aging and possible neurodegeneration may affect memory performance over such a period, independent of epilepsy or surgery. Although our cohort is relatively young (mean age at last follow‐up = 49.3, SD = 8.4), the absence of a healthy age‐matched control group limits our ability to separate surgery‐related decline from normal aging. Furthermore, the use of historical preoperative memory test scores, which were collected up to 2 decades earlier, may not account for improvements in neuropsychological assessment techniques over time. Another limitation is the lack of additional testing to certify hemispheric dominance, which may have an impact on the full grasp of the differences between dominant and nondominant hemispheres. This may be addressed in future studies by incorporating language dominance more consistently when analyzing cognitive outcomes. Lastly, although the RCI helps mitigate practice effects, it does not entirely eliminate the potential for other confounding variables, such as emotional status and interevaluator variability, to influence memory performance outcomes.

## 5 CONCLUSIONS

In summary, our study on a homogeneous sample of patients with TLE/HS, all operated on by the same surgeon and seizure‐free for >10 years, reveals intriguing dynamics in memory function. Memory performance was influenced by preoperative, EEG, demographic, and surgical variables. Although there is initial preservation or improvement in verbal and nonverbal memory function in the first few years postsurgery, there is a tendency for long‐term decline. The extent to which this decline translates into subjective memory complaints remains unclear, particularly because quality of life does not appear to correlate with changes in memory scores.

## AUTHOR CONTRIBUTIONS

Design, data collection, management, analysis and interpretation of the data, as well as preparation, review and approval of the manuscript were under the control and responsibility of the authors. Roberta Gomes, Mirna Portuguez, and Andre Palmini designed the study, coordinated data collection, and wrote the first draft of the manuscript. Eliseu Paglioli, Wyllians Vendramini Borelli, and William Alves Martins obtained demographic and epileptological data and made contributions to the manuscript. Roberta Gomes and Eduardo Leal‐Conceição obtained and interpreted all neuropsychological data and wrote parts of the manuscript. Eliseu Paglioli, Rafael Paglioli, Thomas Frigeri, Wylliams Vendramini Borelli and Williams Alves Martins obtianed demogrpahic and epilepsological data and made contributions to the manuscript.

## CONFLICT OF INTEREST STATEMENT

None of the authors has any conflict of interest to disclose. We confirm that we have read the Journal's position on issues involved in ethical publication and affirm that this report is consistent with those guidelines.

## Data Availability

The data that support the findings of this study are available on request from the corresponding author.
